# Combined *P*‐Value Functions for Compatible Effect Estimation and Hypothesis Testing in Drug Regulation

**DOI:** 10.1002/sim.70224

**Published:** 2025-10-23

**Authors:** Samuel Pawel, Małgorzata Roos, Leonhard Held

**Affiliations:** ^1^ Epidemiology, Biostatistics and Prevention Institute (EBPI), Center for Reproducible Science (CRS) University of Zurich Zurich Switzerland

**Keywords:** confidence interval, estimand, median estimate, meta‐analysis, two‐trials rule

## Abstract

The two‐trials rule in drug regulation requires statistically significant results from two pivotal trials to demonstrate efficacy. However, it is unclear how the effect estimates from both trials should be combined to quantify the drug effect. Fixed‐effect meta‐analysis is commonly used but may yield confidence intervals that exclude the value of no effect even when the two‐trials rule is not fulfilled. We systematically address this by recasting the two‐trials rule and meta‐analysis in a unified framework of combined *p*‐value functions, where they are variants of Wilkinson's and Stouffer's combination methods, respectively. This allows us to obtain compatible combined *p*‐values, effect estimates, and confidence intervals, which we derive in closed‐form. Additionally, we provide new results for Edgington's, Fisher's, Pearson's, and Tippett's *p*‐value combination methods. When both trials have the same true effect, all methods can consistently estimate it, although some show bias. When true effects differ, the two‐trials rule and Pearson's method are conservative (converging to the less extreme effect), Fisher's and Tippett's methods are anti‐conservative (converging to the more extreme effect), and Edgington's method and meta‐analysis are balanced (converging to a weighted average). Notably, Edgington's confidence intervals always asymptotically include the individual trial effects, while meta‐analytic confidence intervals shrink to a point at the weighted average effect. We conclude that all of these methods may be appropriate depending on the estimand of interest. We implement combined *p*‐value function inference for two trials in the R package twotrials, allowing researchers to easily perform compatible hypothesis testing and effect estimation.

## 1 Introduction

The “two‐trials rule” in drug regulation requires “*at least two adequate and well‐controlled studies, each convincing on its own*,” for the demonstration of drug efficacy and subsequent market approval ([1], p. 3). This criterion reflects the need for “substantiation” and “replication” of scientific results ([2], p. 8), and is typically implemented by requiring the *p*‐values from the two trials to be statistically significant at the conventional (one‐sided) α=0.025 level. However, this procedure alone does not provide a combined effect estimate nor a confidence interval (CI), and it has been suggested to pool the estimates with fixed‐effect meta‐analysis for this purpose [3, 4, 5]. Yet, the meta‐analytic CI and point estimate are not always compatible with the two‐trials rule. The meta‐analytic CI may exclude the null value while the two‐trials rule is not fulfilled, leading to discrepancies that are difficult to interpret and communicate.

The results from the two RESPIRE trials [6, 7, 8] in Table 1 illustrate this phenomenon. While the *p*‐value for the null hypothesis of no effect from RESPIRE 1 is p=0.004<0.025, the *p*‐value from RESPIRE 2 is p=0.144>0.025. Hence, the two‐trials rule is not fulfilled at α=0.025. At the same time, the 95% CI for the log rate ratio based on combining the trials' log rate ratio effect estimates with fixed‐effect meta‐analysis ranges from −0.58 to −0.08 and thus excludes the value of 0.

**TABLE 1:** Results from the RESPIRE trials regarding the effect of ciprofloxacin after 14 days for the treatment of non‐cystic fibrosis bronchiectasis [6, 7, 8].

	Log rate ratio	Confidence interval (95%)	*P*‐value (one‐sided)
RESPIRE 1	−0.49	−0.85 to −0.13	0.004
RESPIRE 2	−0.18	−0.53 to 0.16	0.144
Meta‐analysis	−0.33	−0.58 to −0.08	0.004

A first attempt at resolving the apparent paradox could be to realize that the confidence level of the CI does not align with the level of the implicit test underlying the two‐trials rule. Since the two‐trials rule decision is based on two independent tests at level α=0.025, the overall test is at level $\alpha^{2}=0.000625$, thus one could instead take a $(1-2\;\alpha^{2})\times 100\%=99.875\%$ meta‐analytic CI [9, 10]. For the RESPIRE trials, this would lead to a meta‐analytic 99.875% CI from −0.71 to 0.05, which includes the value of 0 and hence aligns with the two‐trials rule decision. However, the level α=0.025 is arbitrary, and it would be desirable to have a CI that is compatible with the two‐trials rule for any level, which is still not the case. For example, for α=0.05, the two‐trials rule is still not fulfilled, while the $(1-2\;\alpha^{2})\times 100\%=99.5\%$ meta‐analytic CI from −0.66 to −0.01 excludes zero.

Despite the widespread use of the two‐trials rule in regulatory decision‐making [11], it remains unclear how point and interval estimation should be reconciled with it. This paper aims to resolve this issue with a new approach. The key idea is to look at both the two‐trials rule and meta‐analysis from the perspective of *p*‐value functions [12, 13, 14, 15, 16] and *p*‐value combination methods [17, 18, 19, 20, 21]. The two‐trials rule can be understood as a combined *p*‐value function based on the squared maximum of two *p*‐values [22] which is a special case of Wilkinson's combination method [23], while meta‐analysis corresponds to the combined *p*‐value function based on Stouffer's *p*‐value combination method [24] with suitable weights. Both can be used to obtain combined *p*‐values for the null hypothesis of no effect, CIs, and point estimates. These quantities are compatible in the sense that the (two‐sided) *p*‐value for a null value is less than α if and only if the null value is excluded by the (1−α)×100% CI, and that the point estimate is included in the CI at any confidence level (1−α)∈(0,1). However, as we will show, the two methods implicitly target different estimands, which explains their different behaviors, and highlights the need to choose the method depending on the scientific question and corresponding estimand of interest. Moreover, the combined *p*‐value function pespective suggests considering alternative *p*‐value combination methods, for example, Edgington's method, based on the sum of *p*‐values [25] or Fisher's method based on the product of *p*‐values [26]. All these *p*‐value combination methods have been studied before in terms of hypothesis testing properties, such as admissibility or monotonicity [17, 27]. In this paper, we take an alternative estimation perspective motivated by practical issues in drug regulation.

This paper is organized as follows: We begin by summarizing the general theory of combined *p*‐value functions (Section 2), followed by investigating combined *p*‐value functions based on the two‐trials rule (Section 2.1), meta‐analysis (Section 2.2), Tippett's method (Section 3.1), Fisher's and Pearson's methods (Section 3.2), and Edgington's method (Section 3.3) in more detail. For each, we derive corresponding point and interval estimates and investigate their properties. Results from two pairs of clinical trials are analyzed to illustrate the characteristics of the methods (Section 4). Extensions to more than two trials are discussed in Section 5. The paper ends with concluding discussions, limitations, and an outlook for future research (Section 6). Appendix A illustrates our R package twotrials for conducting *p*‐value function inference, while Appendix B provides additional technical details.

## 2 Combined *p*‐Value Functions

Suppose that two trials yield the effect estimates ${\hat{\theta }}_{1}$ and ${\hat{\theta }}_{2}$ with corresponding standard errors $\sigma_{1}$ and $\sigma_{2}$, each estimate quantifying the effect of the treatment in the corresponding trial. Typically, it is reasonable to assume that the effect estimates (after suitable transformation) are approximately normally distributed around the trial‐specific true effects $\theta_{1}$ and $\theta_{2}$ with variance equal to their squared standard error, that is, ${\hat{\theta }}_{i}|\theta_{i}∼N(\theta_{i},\sigma_{i}^{2})$ for i∈{1,2}. One‐sided *p*‐values can then be computed by

(1) $$p_{i}(\mu )=\left\{\begin{array}{ll} 1-\Phi (Z_{i})\; & \text{for}\;H_{1i}:\theta_{i}>\mu \;\text{(alternative = “greater”)}\\ \Phi (Z_{i})\; & \text{for}\;H_{1i}:\theta_{i}<\mu \;\text{(alternative = “less”)} \end{array}\right.$$

with z‐values

$$Z_{i}=\frac{{\hat{\theta }}_{i}-\mu }{\sigma_{i}}$$

cumulative distribution function of the standard normal distribution Φ(·), null value μ, and alternative hypothesis $H_{1i}$ chosen based on the orientation of the effect. For example, if a positive effect indicates treatment benefit, the alternative “greater” would be chosen. We will not consider *p*‐values with two‐sided alternatives here, as the hypotheses tested in clinical trials usually have a well‐defined direction. Moreover, combined *p*‐value functions based on two‐sided *p*‐values can behave irregularly, for example, they can be non‐monotone, so that the resulting confidence sets consist of empty or disjoint intervals, which is unintuitive and hard to communicate [28].

A combined *p*‐value function p(μ) is then defined by the function g

$$p(\mu )=g\left(p_{1}(\mu ),p_{2}(\mu )\right)$$

which combines the individual *p*‐value functions $p_{1}(\mu )$ and $p_{2}(\mu )$ into a *p*‐value function p(μ), which is a valid *p*‐value function in the sense of having a uniform distribution for a particular μ if both $p_{1}(\mu )$ and $p_{2}(\mu )$ are also uniformly distributed for that μ [13, 28]. A two‐sided (1−α)×100% CI can then be obtained by determining the null values μ for which the *p*‐value function is equal to α/2 and 1−α/2. The so‐called median estimate is given by the null value μ for which the *p*‐value function equals 1/2 [29]. To obtain these quantities, it is useful to define a “combined estimation function”

$$\hat{\mu }(a)=\left\{\mu :p(\mu )=a\right\}$$

which is the inverse of the combined *p*‐value function. It returns the median estimate when setting a=1/2, while the limits of a (1−α)×100% CI are obtained from a=α/2 and a=1−α/2, respectively. As we will show, combined estimation functions (and hence the median estimate and any CI) are available in closed‐form for several combined *p*‐value functions, including the two‐trials rule and meta‐analysis.

**FIGURE 1:**
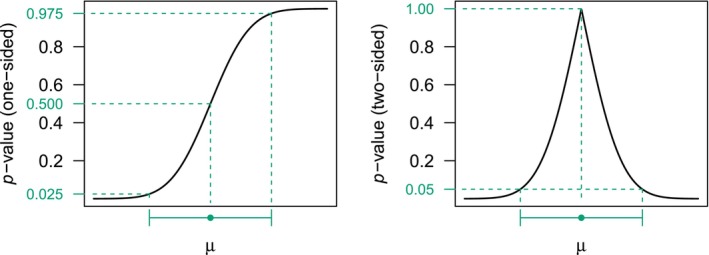
Illustration of a one‐sided *p*‐value function with alternative = “greater” (left plot), corresponding two‐sided *p*‐value function (right plot), and corresponding 95% CI and median estimate.

In practice, it is informative to plot the *p*‐value function for a range of null values μ, see the left plot in Figure 1. For this purpose, it may also be converted to a two‐sided *p*‐value function using the transformation 2min{p(μ),1−p(μ)}, known as “centrality function” [13]. Such a two‐sided *p*‐value function then peaks at the median estimate, and it can be thresholded at α to conveniently read off the (1−α)×100% CI [28], see the right plot in Figure 1.

When both trials have the same underlying true effect ($\theta_{1}=\theta_{2}=\theta$), sometimes called “one population assumption” or “homogeneity” [30, 31], a CI based on a combined *p*‐value function has correct coverage, and the median estimate is median unbiased for the true common effect θ, that is, the probability of the median estimate being greater than θ is equal to the probability of it being smaller than θ (see, e.g., Xie and Singh [13]). However, it is unclear how other operating characteristics (e.g., mean bias or CI width) behave for different combined *p*‐value functions g, and how they behave when the true effects are not the same ($\theta_{1}\neq \theta_{2}$), known as “two populations assumption” or “heterogeneity” [30, 31]. In the following, we will investigate this in detail for the two‐trials rule, meta‐analysis, and four other types of combined *p*‐value functions. As these investigations are somewhat technical, readers may choose to look only at the summary in Table 2 and then jump directly to the applications in Section 4.

**TABLE 2:** Summary of combined *p*‐value functions and corresponding estimation functions. All are based on the alternative “greater”. The median estimate is obtained from setting a=1/2, while the limits of a (1−α)×100% confidence interval (CI) are obtained from a=α/2 and a=1−α/2, respectively.

Method	Combined *p*‐value function	Combined estimation function	Properties
**Two‐trials rule** Maximum *p*‐value, special case of Wilkinson's method, Section 2.1	$p_{\text{2TR}}(\mu )=\max{\left\{p_{1}(\mu ),p_{2}(\mu )\right\}}^{2}$ R function twotrials::p2TR	${\hat{\mu }}_{\text{2TR}}(a)=\min\{{\hat{\theta }}_{1}+\sigma_{1}\;z_{\sqrt{a}},{\hat{\theta }}_{2}+\sigma_{2}\;z_{\sqrt{a}}\}$ R function twotrials::mu2TR	– Targets the least extreme true effect (conservative) – Mean‐biased when trials have the same true effects – CI shrinks to a point with decreasing standard errors – Median estimate not equal to observed effect estimates when the same estimates are obtained in both trials – Median estimate standard error can be larger than trial standard errors
**Fixed‐effect meta‐analysis** Weighted Stouffer's method, inverse‐normal method, Section 2.2	$p_{\text{MA}}(\mu )=1-\Phi (Z_{\text{MA}})$ with $Z_{\text{MA}}=\frac{\Phi^{-1}\{1-p_{1}(\mu )\}/\sigma_{1}+\Phi^{-1}\{1-p_{2}(\mu )\}/\sigma_{2}}{\sqrt{1/\sigma_{1}^{2}+1/\sigma_{2}^{2}}}$ R function twotrials::pMA	${\hat{\mu }}_{\text{MA}}(a)={\hat{\theta }}_{\text{MA}}+\sigma_{\text{MA}}\;z_{a}$ with $\begin{array}{cc} \sigma_{\text{MA}}^{2} & =1/(1/\sigma_{1}^{2}+1/\sigma_{2}^{2})\\ {\hat{\theta }}_{\text{MA}} & =({\hat{\theta }}_{1}/\sigma_{1}^{2}+{\hat{\theta }}_{2}/\sigma_{2}^{2})\;\sigma_{\text{MA}}^{2} \end{array}$ R function twotrials::muMA	– Targets weighted average effect (inverse squared standard error weights) – Mean‐unbiased when the same true effects – CI shrinks to a point with decreasing standard errors – Median estimate equals observed effect estimates when the same estimates are observed in both trials – Median estimate standard error cannot be larger than trial standard errors
**Tippett's method** Minimum *p*‐value, special case of Wilkinson's method, Section 3.1	$p_{T}(\mu )=1-{\left(1-\min\{p_{1}(\mu ),p_{2}(\mu )\}\right)}^{2}$ R function twotrials::pTippett	${\hat{\mu }}_{T}(a)=\max\{{\hat{\theta }}_{1}-\sigma_{1}\;z_{\sqrt{1-a}},{\hat{\theta }}_{2}-\sigma_{2}\;z_{\sqrt{1-a}}\}$ R function twotrials::muTippett	– Targets most extreme true effect (anti‐conservative) – Mean‐biased when the same true effects – CI shrinks to a point with decreasing standard errors – Median estimate not equal to observed effect estimates when the same estimates are observed in both trials – Median estimate standard error can be larger than trial standard errors
**Fisher's method** Product of *p*‐values, Section 3.2	$p_{F}(\mu )=1-\mathrm{Pr}(\chi_{4}^{2}\leq F)$ with $F=-2[\log\{p_{1}(\mu )\}+\log\{p_{2}(\mu )\}]$ R function twotrials::pFisher	${\hat{\mu }}_{F}(a)$ not analytically available R function twotrials::muFisher	– Targets most extreme true effect (anti‐conservative) – CI shrinks to a point with decreasing standard errors – Median estimate not equal to observed effect estimates when the same estimates are observed in both trials – Median estimate standard error can be larger than trial standard errors
**Pearson's method** Product of 1−p‐values, Section 3.2	$p_{P}(\mu )=\mathrm{Pr}(\chi_{4}^{2}\leq K)$ with $K=-2[\log\{1-p_{1}(\mu )\}+\log\{1-p_{2}(\mu )\}]$ R function twotrials::pPearson	${\hat{\mu }}_{P}(a)$ not analytically available R function twotrials::muPearson	– Targets the least extreme true effect (conservative) – CI shrinks to a point with decreasing standard errors – Median estimate not equal to observed effect estimates when the same estimates are observed in both trials – Median estimate standard error can be larger than trial standard errors
**Edgington's method** Sum of *p*‐values, Section 3.3	$\begin{array}{c} p_{E}(\mu )=\left\{\begin{array}{ll} E^{2}/2\; & \text{if}\;0\leq E\leq 1\\ 1-{\left(2-E\right)}^{2}/2\; & \text{if}\;1<E\leq 2 \end{array}\right. \end{array}$ $\begin{array}{c} \text{with}\;E=p_{1}(\mu )+p_{2}(\mu )\; \end{array}$ R function twotrials::pEdgington	Median estimate analytically available ${\hat{\mu }}_{E}(a=1/2)=\frac{{\hat{\theta }}_{1}/\sigma_{1}+{\hat{\theta }}_{2}/\sigma_{2}}{1/\sigma_{1}+1/\sigma_{2}}$ ${\hat{\mu }}_{E}(a)$ not analytically available for a≠1/2 R function twotrials::muEdgington	– Targets weighted average effect (inverse standard error weights) – Mean‐unbiased when the same true effects – CI asymptotically always includes both true effects (only shrinks to a point when both are equal) – Median estimate equals observed effect estimates when the same estimates are observed in both trials – Median estimate standard error can be larger than trial standard errors

### 2.1 The Two‐Trials Rule (Maximum Method)

The two‐trials rule is fulfilled if $\max\{p_{1},p_{2}\}\leq \alpha$, or equivalently if

(2) $$\begin{array}{l} p_{\text{2TR}}(\mu )=\max{\left\{p_{1}(\mu ),p_{2}(\mu )\right\}}^{2}\leq \alpha^{2} \end{array}$$

The formulation using the squared maximum (2) may be preferable because $p_{\text{2TR}}(\mu )$ is a valid *p*‐value, that is, it has a uniform distribution if both $p_{1}(\mu )$ and $p_{2}(\mu )$ are also uniformly distributed for a particular μ [22]. The combined *p*‐value function (2) is also a special case of Wilkinson's *p*‐value combination method based on the rth smallest out of k *p*‐values with r=k=2 [23]. This relationship can be used to generalize the two‐trials rule to different settings while preserving type I error control at level $\alpha^{2}$, for example, settings with three rather than two trials [32]. We will discuss such extensions to more than two trials in Section 5 and focus first on effect estimation for two trials.

#### 2.1.1 Effect Estimation

To obtain a CI and a point estimate based on the two‐trials rule, we can equate the combined *p*‐value function (2) to some value a∈(0,1) and solve for the null value μ. This leads to the combined estimation function

(3) $${\hat{\mu }}_{\text{2TR}}(a)=\left\{\begin{array}{ll} \min\{{\hat{\theta }}_{1}+\sigma_{1}\;z_{\sqrt{a}},{\hat{\theta }}_{2}+\sigma_{2}\;z_{\sqrt{a}}\}\; & \;\text{for alternative = “greater”}\\ \;\max\{{\hat{\theta }}_{1}-\sigma_{1}\;z_{\sqrt{a}},{\hat{\theta }}_{2}-\sigma_{2}\;z_{\sqrt{a}}\} & \text{for alternative = “less”} \end{array}\right.$$

with $z_{q}$ the q×100% quantile of the standard normal distribution. For a=1/2, the median estimate is obtained, while the limits of an (1−α)×100% CI can be obtained from a=α/2 and a=1−α/2.

Now assume that the standard errors of both trials are the same ($\sigma_{1}=\sigma_{2}=\sigma$) and the alternative is “greater”. The median estimate is then

(4) $${\hat{\mu }}_{\text{2TR}}(1/2)=\min\{{\hat{\theta }}_{1},{\hat{\theta }}_{2}\}+\sigma \;\underset{0.54}{\underbrace{z_{\sqrt{1/2}}}}$$

and the 95% CI is given by

(5) $$\left[\min\{{\hat{\theta }}_{1},{\hat{\theta }}_{2}\}+\sigma \;\underset{-1}{\underbrace{z_{\sqrt{0.025}}}},\;\min\{{\hat{\theta }}_{1},{\hat{\theta }}_{2}\}+\sigma \;\underset{2.24}{\underbrace{z_{\sqrt{0.975}}}}\right]$$

**FIGURE 2:**
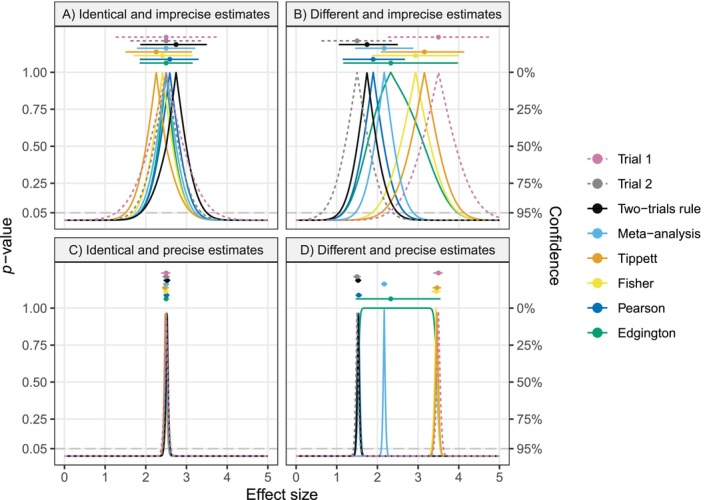
Four hypothetical pairs of effect estimates and standard errors from two trials. The standard errors are assumed to be of the form $\sigma =\sqrt{2/n}$. The sample size n in trial 1 is set to 5 (imprecise) or 500 (precise), and in trial 2 to twice the sample size of trial 1. The two‐sided *p*‐value functions of the individual trials (dashed lines), and the combined *p*‐value functions (solid lines) based on the two‐trials rule, fixed‐effect meta‐analysis, Tippett's, Fisher's, Pearson's, and Edgington's methods are shown along with the corresponding 95% CIs and median estimates (top). All *p*‐values are based on the alternative “greater” and then converted to two‐sided *p*‐values via the centrality function 2min{p,1−p}.

Both seem counterintuitive. For instance, if the trial effect estimates are the same (${\hat{\theta }}_{1}={\hat{\theta }}_{2}=\hat{\theta }$), the median estimate (4) is shifted away from the observed estimate by $\sigma \times z_{\sqrt{1/2}}\approx \sigma \times 0.54$, and also the CI (5) is not centered around it. This is illustrated in Figure 2 (panels A and C), where the hypothetical trial effect estimates are identical, but the median estimates based on the two‐trials rule (black) are larger. Moreover, the CI (5) is skewed in the sense that the distance between the upper limit and the median estimate is larger than the distance between the lower limit and the median estimate although the estimates are the same.

While this CI has correct coverage, and the median estimate is median unbiased, we may look at other operating characteristics. The expectation of the median estimate (4) can be derived to be

(6) $$\begin{array}{ll} E\left[{\hat{\mu }}_{\text{2TR}}(1/2)\right] & =\theta_{1}\;\Phi \left(\frac{\theta_{2}-\theta_{1}}{\sqrt{2}\;\sigma }\right)+\theta_{2}\;\Phi \left(\frac{\theta_{1}-\theta_{2}}{\sqrt{2}\;\sigma }\right)\\ & \;+\sigma \;\left\{z_{\sqrt{1/2}}-\sqrt{2}\;\phi \;\left(\frac{\theta_{2}-\theta_{1}}{\sqrt{2}\;\sigma }\right)\right\}\; \end{array}$$

where ϕ(·) denotes the density function of the standard normal distribution, see Appendix B for details. If the true effects from the two trials coincide ($\theta_{1}=\theta_{2}=\theta$) the first two terms of the expectation (6) reduce to the common effect θ, whereas the last term reduces to $\sigma \times (z_{\sqrt{1/2}}-1/\sqrt{\pi })\approx \sigma \times$ −0.019. Hence, the median estimate with (4) is negatively biased, yet the bias vanishes as the standard error decreases. Similarly, one can show that the median estimate for the alternative “less” is positively biased, so the median estimate from the two‐trials rule exhibits a conservative bias in both cases.

Another interesting operating characteristic is the standard error of the median estimate. An intuitively desirable property is that the standard error of a combined estimate should not be larger than either of the trials' standard errors. Assuming again that the true trial effects and standard errors coincide, the two‐trials rule satisfies this, as the standard error takes the simple form

(7) $$\sigma_{\text{2TR}}=\sigma \sqrt{1-1/\pi }\approx \sigma \times 0.83$$

so it is approximately 17% smaller than the standard errors of each individual trial. However, this is no longer the case when the trial standard errors differ, where the standard error of the two‐trial rule also depends on the true trial effects. See Appendix B for more details, also on standard errors based on other combined *p*‐value functions.

#### 2.1.2 Asymptotics

Suppose now that the sample size of the trials increases, and in turn the standard errors of the effect estimates decrease toward zero. The combined estimation function (3) then converges to

$$\begin{array}{l} \underset{\sigma_{1},\sigma_{2}↓0}{\text{plim}}{\hat{\mu }}_{\text{2TR}}(a)=\left\{\begin{array}{ll} \min\{\theta_{1},\theta_{2}\}\; & \;\text{for alternative = “greater”}\\ \;\max\{\theta_{1},\theta_{2}\} & \text{for alternative = “less”} \end{array}\right. \end{array}$$

see Appendix B for details. Hence, the median estimate (a=1/2) and any CI limit (a≠1/2) approach $\min\{{\hat{\theta }}_{1},{\hat{\theta }}_{2}\}$ or $\max\{{\hat{\theta }}_{1},{\hat{\theta }}_{2}\}$, depending on the alternative hypothesis. This means that the CI shrinks to the more conservative of the two effects, while in case they coincide ($\theta_{1}=\theta_{2}=\theta$) it shrinks to the common effect θ. Both scenarios are illustrated in Figure 2: In the case of very small standard errors and different effect estimates (panel D), the CI based on the two‐trials rule (black) is tightly concentrated around the smaller effect estimate, while for identical effect estimates (panel C), it is tightly concentrated around the common effect estimate.

### 2.2 Fixed‐Effect Meta‐Analysis (Stouffer's Method)

We will now compare the *p*‐value function from the two‐trials rule with its meta‐analysis counterpart. The combined *p*‐value based on fixed‐effect meta‐analysis is given by

(8) $$\begin{array}{l} p_{\text{MA}}(\mu )=\left\{\begin{array}{ll} 1-\Phi (Z_{\text{MA}})\; & \text{for alternative = “greater”}\\ \Phi (Z_{\text{MA}})\; & \text{for alternative = “less”} \end{array}\right. \end{array}$$

with

(9) $$\begin{array}{l} Z_{\text{MA}}=\frac{Z_{1}/\sigma_{1}+Z_{2}/\sigma_{2}}{\sqrt{1/\sigma_{1}^{2}+1/\sigma_{2}^{2}}}=\frac{{\hat{\theta }}_{\text{MA}}-\mu }{\sigma_{\text{MA}}} \end{array}$$

where

(10) $$\begin{array}{l} {\hat{\theta }}_{\text{MA}}=\frac{{\hat{\theta }}_{1}/\sigma_{1}^{2}+{\hat{\theta }}_{2}/\sigma_{2}^{2}}{1/\sigma_{1}^{2}+1/\sigma_{2}^{2}} \end{array}$$

and

(11) $$\begin{array}{l} \sigma_{\text{MA}}=\frac{1}{\sqrt{1/\sigma_{1}^{2}+1/\sigma_{2}^{2}}} \end{array}$$

The first equation in Equation (9) represents Stouffer's *p*‐value combination method (after transforming *p*‐values to z‐values) using inverse standard errors as weights [19], whereas the second equation in Equation (9) shows the corresponding representation via the meta‐analytically pooled estimate (10) and standard error (11) [33]. While meta‐analytic pooling could be extended to the random‐effects model, this is typically not desired with only two studies for three reasons. First, the interest is in the true effects underlying the studies. Second, random‐effects variance estimation is unreliable with only two studies. Finally, even if there is effect heterogeneity, fixed‐effect meta‐analysis is a valid procedure that estimates a well‐defined average true effect [34].

#### 2.2.1 Effect Estimation

To obtain meta‐analytic CIs and point estimates, we can also equate the *p*‐value function (8) to a and solve for μ. This leads to the combined estimation function

$$\begin{array}{l} {\hat{\mu }}_{\text{MA}}(a)=\left\{\begin{array}{ll} {\hat{\theta }}_{\text{MA}}+\sigma_{\text{MA}}\;z_{a}\; & \text{for alternative = “greater”}\\ {\hat{\theta }}_{\text{MA}}-\sigma_{\text{MA}}\;z_{a}\; & \text{for alternative = “less”} \end{array}\right. \end{array}$$

When a=1/2 we obtain ${\hat{\theta }}_{\text{MA}}$ as the median estimate, while a=α/2 and a=1−α/2 give the limits of the (1−α)×100% CI corresponding to the usual fixed‐effect meta‐analytic Wald CI.

The standard error (11) of the meta‐analytic median estimate has two desirable properties: First, it is never larger than either of trials' standard errors ($\sigma_{\text{MA}}\leq \min\{\sigma_{1},\sigma_{2}\}$). Second, under effect homogeneity, the standard error is the smallest among all unbiased estimators of the common effect [17]. Both properties fail to hold for the two‐trials rule and the other *p*‐value combination methods discussed below.

#### 2.2.2 Asymptotics

Since the meta‐analytic combined estimation function is a linear combination of normally distributed effect estimates, its distribution is also normal and given by

$$\begin{array}{l} {\hat{\mu }}_{\text{MA}}(a)∼N\left(\frac{\theta_{1}}{1+c}+\frac{\theta_{2}}{1+1/c}-\frac{z_{a}}{\sqrt{1/\sigma_{1}^{2}+1/\sigma_{2}^{2}}},\;\frac{1}{1/\sigma_{1}^{2}+1/\sigma_{2}^{2}}\right) \end{array}$$

for the alternative “greater” and with variance ratio $c=\sigma_{1}^{2}/\sigma_{2}^{2}$. For the alternative “less”, the minus in the mean has to be replaced with a plus. The median estimate (a=1/2) hence targets the weighted average of the true effects

$$\begin{array}{l} \frac{\theta_{1}}{1+c}+\frac{\theta_{2}}{1+1/c} \end{array}$$

while the meta‐analytic CIs become increasingly concentrated around the weighted average with decreasing standard errors, provided the relative variance c stays constant.

Meta‐analysis thus shows a less conservative asymptotic behavior than the two‐trials rule in the sense that a more extreme effect can compensate for a less extreme one, whereas the two‐trials rule would converge to the less extreme of the two effects. Figure 2 illustrates this asymptotic behavior: In case both estimates are identical and the standard errors are very small (panel C), the meta‐analytic CI is concentrated around the trials estimate, while in the case of different estimates, the CI concentrates somewhere in between (panel D). Since in this example the relative variance is c=2, the weighted average is slightly closer to the estimate from trial 2.

## 3 Other *p*‐Value Combination Methods

While the two‐trials rule and meta‐analysis are the most commonly used *p*‐value combination methods in practice, several other combination methods exist [17]. In this section, we examine Tippett's, Fisher's, Pearson's, and Edgington's methods, which can also be used to obtain combined effect estimates, CIs, and *p*‐values. Although these methods are not standard in drug regulation, they may have useful properties in certain settings, as we will demonstrate in the following.

### 3.1 Tippett's (Minimum) Method

The combined *p*‐value from Tippett's method [35] is based on the minimum of the two *p*‐values and given by

$$p_{T}(\mu )=1-{\left(1-\min\{p_{1}(\mu ),p_{2}(\mu )\}\right)}^{2}$$

It is closely related to the two‐trials rule in the sense that the combined *p*‐value based on the alternative “greater” from Tippett's method is the same as one minus the combined *p*‐value based on the alternative “less” from the two‐trials rule, and vice versa [28]. Similarly, Tippett's method is a special case of Wilkinson's method based on the r=1 smallest out of k=2 *p*‐values.

#### 3.1.1 Effect Estimation

Following a similar approach to that the two‐trials rule, CIs and point estimates based on Tippett's method can be obtained in closed‐form with the combined estimation function

(12) $${\hat{\mu }}_{T}(a)=\left\{\begin{array}{ll} \;\max\{{\hat{\theta }}_{1}-\sigma_{1}\;z_{\sqrt{1-a}},{\hat{\theta }}_{2}-\sigma_{2}\;z_{\sqrt{1-a}}\}\; & \text{for alternative = “greater”}\\ \min\{{\hat{\theta }}_{1}+\sigma_{1}\;z_{\sqrt{1-a}},{\hat{\theta }}_{2}+\sigma_{2}\;z_{\sqrt{1-a}}\}\; & \;\text{for alternative = “less”} \end{array}\right.$$

The similarity to the two‐trials rule is again visible as (12) looks similar to the estimation function from the two‐trials rule (3) with the minimum and maximum flipped and using different normal quantiles. In particular, the same median estimates are obtained (i.e., ${\hat{\mu }}_{\text{2TR}}(1/2)={\hat{\mu }}_{T}(1/2)$) if opposite alternatives are specified.

We can see that when the observed effect estimates are the same (${\hat{\theta }}_{1}={\hat{\theta }}_{2}=\hat{\theta }$), the median estimate (a=1/2) based on Tippett's method is not equal to $\hat{\theta }$ but shifted from it, as the two‐trials rule (see panels A and C in Figure 2 for an illustration). Similarly, CIs obtained from Tippett's method are typically skewed in the sense that the distances between the point estimate and the upper and lower limits are not the same.

#### 3.1.2 Asymptotics

It can be shown that as the standard errors $\sigma_{1}$ and $\sigma_{2}$ decrease, the combined estimation function (12) converges to

$$\begin{array}{l} \underset{\sigma_{1},\sigma_{2}↓0}{\text{plim}}{\hat{\mu }}_{T}(a)=\left\{\begin{array}{ll} \max\{\theta_{1},\theta_{2}\}\; & \text{for alternative = “greater”}\\ \min\{\theta_{1},\theta_{2}\}\; & \;\text{for alternative = “less”} \end{array}\right. \end{array}$$

that is, the more extreme of the two effects, see Appendix B for details. In contrast to the two‐trials rule, Tippett's method is hence anti‐conservative. This is illustrated in panel D of Figure 2, where Tippett's CI is tightly concentrated around the larger effect estimate.

### 3.2 Fisher's and Pearson's (Product) Methods

Pearson's and Fisher's combination method are two closely related *p*‐value combination methods that are based on the product of *p*‐values, or equivalently, the sum of the log *p*‐values. Fisher's method has been proposed for combining *p*‐values from clinical trials [9, 30, 36], however, using the associated *p*‐value function for effect estimation in a regulatory trials setting has remained unexplored.

The combined *p*‐value function based on Fisher's method [26] is given by

(13) $$p_{F}(\mu )=1-\mathrm{Pr}\left(\chi_{4}^{2}\leq -2[\log\{p_{1}(\mu )\}+\log\{p_{2}(\mu )\}]\right)$$

while the combined *p*‐value function based on Pearson's method [37] is given by

(14) $$p_{P}(\mu )=\mathrm{Pr}\left(\chi_{4}^{2}\leq -2[\log\{1-p_{1}(\mu )\}+\log\{1-p_{2}(\mu )\}]\right)$$

Pearson [38] proposed also another method based on the maximum of the test statistics underlying the *p*‐values (13) and (14), but we will not consider this method here as its test statistic does not have an exact null distribution [39]. As with the two‐trials rule and Tippett's method, the combined *p*‐value functions of Fisher's and Pearson's methods are related in the sense that the *p*‐value function based on Fisher's method and the alternative hypothesis “greater” is the same as one minus the *p*‐value function based on Pearson's method and the alternative “less”, and vice versa [28]. As we will show in the following, Fisher's method also acts in a similar anti‐conservative way as Tippett's method, while Pearson's method acts in a similar conservative way as the two‐trials rule.

#### 3.2.1 Effect Estimation

CIs and point estimates based on Fisher's and Pearson's methods can in general not be obtained in closed‐form but require numerical root‐finding. However, a special case where a closed‐form solution is available is when the effect estimates and standard errors are the same in both trials (${\hat{\theta }}_{1}={\hat{\theta }}_{2}=\hat{\theta }$ and $\sigma_{1}=\sigma_{2}=\sigma$). This is unrealistic in practice, but it serves as an important soundness check to investigate whether the methods produce reasonable estimates in the situation of identical trial results. In this case, we obtain the following closed‐form combined estimation function for Fisher's method

(15) $${\hat{\mu }}_{F}(a)=\left\{\begin{array}{ll} \hat{\theta }+\sigma \;z_{\exp\{-\chi_{4}^{2}(1-a)/4\}}\; & \text{for alternative = “greater”}\\ \hat{\theta }-\sigma \;z_{\exp\{-\chi_{4}^{2}(1-a)/4\}}\; & \;\text{for alternative = “less”} \end{array}\right.$$

and for Pearson's method

(16) $${\hat{\mu }}_{P}(a)=\left\{\begin{array}{ll} \hat{\theta }-\sigma \;z_{\exp\{-\chi_{4}^{2}(a)/4\}}\; & \text{for alternative = “greater”}\\ \hat{\theta }+\sigma \;z_{\exp\{-\chi_{4}^{2}(a)/4\}}\; & \;\text{for alternative = “less”} \end{array}\right.$$

with $\chi_{4}^{2}(a)$ the a×100% quantile of the chi‐squared distribution with four degrees of freedom. Importantly, the median estimates (a=1/2) from both methods do not equal the observed estimate $\hat{\theta }$ but are shifted away from it by $z_{\exp\{-\chi_{4}^{2}(1/2)/4\}}\approx -0.17$ standard errors σ, similar to the two‐trials rule and Tippett's method. Another similarity is that the CI is skewed since the distance between the lower and upper limits to the point estimate is not the same.

#### 3.2.2 Asymptotics

To understand the asymptotic behavior of Fisher's and Pearson's methods, we may again, examine their combined estimation functions for decreasing the standard errors. When the true effects are equal ($\theta_{1}=\theta_{2}=\theta$), both Fisher's and Pearson's median estimates will converge toward it, which is clear from the theory of *p*‐value functions but can also be informally seen from Equations (15) and (16) shrinking toward the common effect estimate for a decreasing standard error. On the other hand, when the true effects are unequal, it can then be shown that

$$\begin{array}{l} \underset{\sigma_{1},\sigma_{2}↓0}{\text{plim}}{\hat{\mu }}_{F}(a)=\left\{\begin{array}{ll} \max\{\theta_{1},\theta_{2}\}\; & \text{for alternative = “greater”}\\ \min\{\theta_{1},\theta_{2}\}\; & \;\text{for alternative = “less”} \end{array}\right. \end{array}$$

and

$$\begin{array}{l} \underset{\sigma_{1},\sigma_{2}↓0}{\text{plim}}{\hat{\mu }}_{P}(a)=\left\{\begin{array}{ll} \min\{\theta_{1},\theta_{2}\}\; & \text{for alternative = “greater”}\\ \max\{\theta_{1},\theta_{2}\}\; & \;\text{for alternative = “less”} \end{array}\right. \end{array}$$

see Appendix B for details. This means that the combined estimation functions converge toward the more extreme effect for Fisher's method (e.g., the maximum of two positively oriented effects), and the less extreme effect for Pearson's method (e.g., the minimum of two positively oriented effects). The behavior is similar to Tippett's method and the two‐trials rule where one method acts anti‐conservative (Fisher and Tippett's methods), while the other methods act conservatively (Pearson's method and the two‐trials rule). However, the examples in panels B and D of Figure 2 suggest that in finite samples, Fisher's and Pearson's methods remain closer to the weighted average compared to Tippett's method and the two‐trials rule.

### 3.3 Edgington's (Sum) Method

Edgington's method based on the sum of *p*‐values [25, 40] is yet another *p*‐value combination method that can be used for obtaining a combined *p*‐value function, and the last method that we will consider in this paper. It is given by

(17) $$p_{E}(\mu )=\left\{\begin{array}{ll} E^{2}/2\; & \text{if}\;0\leq E\leq 1\\ 1-{\left(2-E\right)}^{2}/2\; & \text{if}\;1<E\leq 2 \end{array}\right.$$

with $E=p_{1}(\mu )+p_{2}(\mu )$. An attractive feature is that two‐sided CIs based on Edgington's method are orientation invariant, which is not the case for the other combined *p*‐value functions considered so far. That is, CIs based on Edgington's method do not depend on the orientation of the underlying one‐sided *p*‐values, so the same CI is obtained regardless of whether one uses *p*‐values with the alternative “greater” or “less” [28]. Edgington's method has previously been used in meta‐analysis [28], to synthesize *p*‐values from original and replication studies [40], and suggested as an alternative for the two‐trials rule [22]. However, its estimation properties in the context of two trials remain unexplored.

#### 3.3.1 Effect Estimation

The median estimate based on Edgington's method has an intuitive interpretation as the null value μ for which the sum of the *p*‐values is one. It can be obtained in closed‐form by

(18) $${\hat{\mu }}_{E}(1/2)=\frac{{\hat{\theta }}_{1}/\sigma_{1}+{\hat{\theta }}_{2}/\sigma_{2}}{1/\sigma_{1}+1/\sigma_{2}}$$

so is a weighted average of the two effect estimates, as the meta‐analytic point estimate (10). However, the weights from Edgington's method are equal to the inverse standard errors, whereas the weights from meta‐analysis are equal to the inverse squared standard errors. Thus, Edgington's method gives more weight to smaller studies (those with larger standard errors) compared to meta‐analysis. Moreover, since the expectation of the median estimate (18) is again a weighted average of the true effects, it follows that Edgington's median estimate is unbiased when the true effects coincide ($\theta_{1}=\theta_{2}=\theta$), while in case they differ, the median estimate targets a weighted average of the true effects, although not the same weighted average as targeted by meta‐analysis.

The standard error of Edgington's median estimate is given by

(19) $$\sigma_{E}=\frac{\sqrt{2}}{1/\sigma_{1}+1/\sigma_{2}}$$

and does not depend on the true effects, similar to meta‐analysis, but unlike the two‐trials rule. It is always larger than the meta‐analytic standard error (11), see Appendix B. Therefore, under effect homogeneity, Edgington's method is less efficient than meta‐analysis at estimating the common effect. Under effect heterogeneity, however, the two methods target different estimands, so a comparison of their standard errors may not be meaningful. Finally, unlike meta‐analysis, Edgington's standard error is not always smaller than or equal to either of the two trials' standard errors. This is only the case if the standard error ratio is $\sqrt{2}-1\leq \sigma_{2}/\sigma_{1}\leq \sqrt{2}+1$. For example, suppose $\sigma_{1}=0.5$ and $\sigma_{2}=2$, then Edgington's standard error is $\sqrt{2}/(2+0.5)=0.566$, which is greater than $\sigma_{1}$.

In general, CIs for Edgington's method do not have closed‐form solutions and must be computed numerically. Nevertheless, as with Pearson's and Fisher's methods, a closed‐form combined estimation function is available when the effect estimates and standard errors from both trials coincide ($\hat{\theta }={\hat{\theta }}_{1}={\hat{\theta }}_{2}$ and $\sigma =\sigma_{1}=\sigma_{2}$), which enables again analytical assessment of how the CI behaves in this important scenario. In this case, the combined estimation function is

(20) $${\hat{\mu }}_{E}(a)=\left\{\begin{array}{ll} \;\hat{\theta }+\sigma \;z_{\sqrt{a/2}}\; & \text{for}\;a\leq 1/2\\ \hat{\theta }-\sigma \;z_{\sqrt{(1-a)/2}}\; & \text{for}\;a>1/2 \end{array}\right.$$

for the alternative “greater” and with the plus (minus) after $\hat{\theta }$ replaced with minus (plus) in Equation (20) for the alternative “less”. We can see that the CIs obtained from Equation (20) are symmetric and centered around the observed effect estimate $\hat{\theta }$, similar to meta‐analysis but unlike the CIs from the two‐trials rule, Tippett's, Fisher's, and Pearson's methods. Yet, Edgington's CI is in this case narrower than the meta‐analytic CI. For example, Edgington's 95% CI is 12.2% narrower than the corresponding meta‐analytic 95% CI. Panel A of Figure 2 illustrates this as Edgington's CI is narrower than the meta‐analytic CI, although both are centered around the same effect estimate. However, in case the trials' effect estimates are different, Edgington's CI can also be much wider. For instance, in panel B of Figure 2 where the trials produced very different results; Edgington's CI is much wider than any of the other methods. This suggests that Edgington's method reacts to heterogeneity by widening its CI to include both trial effect estimates.

#### 3.3.2 Asymptotics

Because the median estimate based on Edgington's method is a weighted average of two normally distributed effect estimates, it is also normally distributed

$$\begin{array}{l} {\hat{\mu }}_{E}(1/2)∼N\left(\frac{\theta_{1}}{1+\sqrt{c}}+\frac{\theta_{2}}{1+1/\sqrt{c}},\;\frac{2}{{\left(1/\sigma_{1}+1/\sigma_{2}\right)}^{2}}\right) \end{array}$$

with relative variance $c=\sigma_{1}^{2}/\sigma_{2}^{2}$. As the median estimate has its mean at the weighted average

$$\begin{array}{l} \frac{\theta_{1}}{1+\sqrt{c}}+\frac{\theta_{2}}{1+1/\sqrt{c}} \end{array}$$

it is clear that it will converge toward it as the standard errors decrease. Whether the CI shrinks to this weighted average depends on whether the true effects are equal. In case they are, it can be informally seen that the CI (20) will shrink to the common true effect, which is illustrated in panel C of Figure 2. However, when the true effects differ, the CI will not shrink to a point, but will remain an interval that always includes both true effects, as the limiting combined estimation function is

(21) $${\text{plim}}_{\sigma_{1},\sigma_{2}↓0}{\hat{\mu }}_{E}(a)=\left\{\begin{array}{ll} \min\{\theta_{1},\theta_{2}\}\; & \text{for}\;a<1/2\\ \frac{\theta_{1}}{1+\sqrt{c}}+\frac{\theta_{2}}{1+1/\sqrt{c}}\; & \text{for}\;a=1/2\\ \max\{\theta_{1},\theta_{2}\}\; & \text{for}\;a>1/2 \end{array}\right.$$

see Appendix B for details. This means that CIs based on Edgington's method will asymptotically always include both true effects, even when the trials' sample sizes become arbitrarily large, see panel D of Figure 2 for an illustration. This behavior is strikingly different from meta‐analysis, whose CI shrinks to a point at the weighted average, even when the true effects are not the same.

## 4 Applications

We will now illustrate combined *p*‐value functions, CIs, and median estimates on data from two different pairs of clinical trials.

### 4.1 The RESPIRE Trials

**FIGURE 3:**
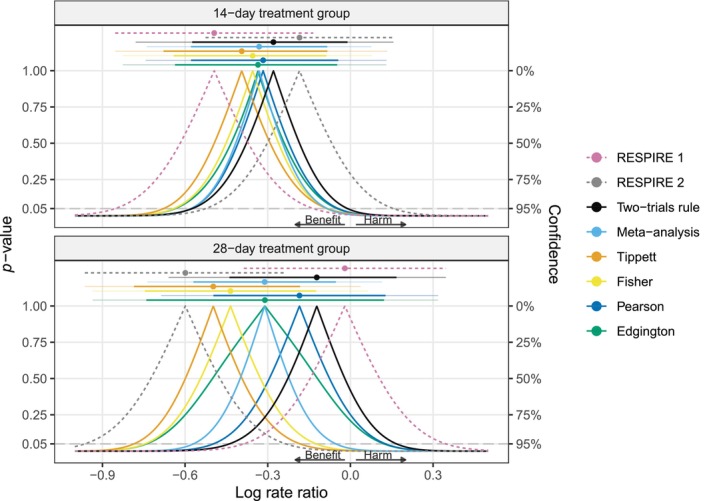
Results of the RESPIRE trials [6, 7, 8] for the effect of ciprofloxacin over 14 days (top) or 28 days (bottom) compared to placebo for the treatment of non‐cystic fibrosis bronchiectasis. The two‐sided *p*‐value functions of the individual trials (dashed lines), and the combined *p*‐value functions (solid lines) based on the two‐trials rule, fixed‐effect meta‐analysis, Tippett's, Fisher's, Pearson's, and Edgington's methods are shown along with corresponding median estimates and CIs (95% and 99.875% via telescope lines). All *p*‐values are based on the alternative “less” and then converted to two‐sided *p*‐values via the centrality function 2min{p,1−p}.

We first revisit the RESPIRE trials [6, 7, 8], which were presented as motivating examples in Table 1 in the introduction. The trials investigated the effect of ciprofloxacin in the treatment of non‐cystic fibrosis bronchiectasis. Each trial had two treatment groups (on/off treatment cycles of either 14 or 28 days for 48 weeks) and two corresponding control groups. RESPIRE 1 showed a substantial treatment effect in the 14‐day treatment regimen (estimated log rate ratio of $\log\hat{\text{RR}}=-0.49$ with 95% CI from −0.85 to −0.13), while the benefit was less pronounced in RESPIRE 2 ($\log\hat{\text{RR}}=-0.18$ with 95% CI from −0.53 to 0.16). Surprisingly, this was reversed for the 28‐day regimens, with RESPIRE 2 showing a much stronger treatment effect ($\log\hat{\text{RR}}=-0.6$ with 95% CI from −0.96 to −0.23) while RESPIRE 1 showed almost no benefit ($\log\hat{\text{RR}}=-0.02$ with 95% CI from −0.39 to 0.35). Figure 3 shows the *p*‐value functions of the two studies (dashed lines) along with different combined *p*‐value functions (solid lines) and corresponding point estimates and CIs (top). Table 3 shows the results in numerical form. Of note, all results were computed with our R package twotrials, and Appendix A shows how the results for the 14‐day treatment group can be reproduced.

**TABLE 3:** Point estimates (with implicit weights), 95% CIs (with widths), and *p*‐values for the RESPIRE trials [6, 7, 8]. Note that weights and CI widths are computed from unrounded numbers and may not always exactly correspond to the rounded point estimates and CIs.

	Log rate ratio	Weight RESPIRE 1	95% CI	CI width	*P*‐value (one‐sided)
*14‐day treatment group*					
RESPIRE 1	−0.49		−0.85 to −0.13	0.72	0.00351
RESPIRE 2	−0.18		−0.53 to 0.16	0.68	0.14400
Two‐trials rule	−0.28	0.31	−0.57 to −0.01	0.56	0.02073
Meta‐analysis	−0.33	0.47	−0.58 to −0.08	0.49	0.00432
Tippett	−0.39	0.68	−0.68 to −0.08	0.59	0.00701
Fisher	−0.35	0.55	−0.64 to −0.09	0.55	0.00434
Pearson	−0.32	0.43	−0.58 to −0.04	0.53	0.01138
Edgington	−0.34	0.49	−0.64 to −0.05	0.59	0.01088
*28‐day treatment group*					
RESPIRE 1	−0.02		−0.39 to 0.35	0.73	0.45699
RESPIRE 2	−0.60		−0.96 to −0.23	0.73	0.00064
Two‐trials rule	−0.12	0.82	−0.44 to 0.17	0.61	0.20884
Meta‐analysis	−0.31	0.50	−0.57 to −0.05	0.52	0.00912
Tippett	−0.50	0.18	−0.79 to −0.18	0.60	0.00127
Fisher	−0.44	0.28	−0.75 to −0.12	0.62	0.00266
Pearson	−0.18	0.72	−0.50 to 0.13	0.62	0.12562
Edgington	−0.31	0.50	−0.74 to 0.12	0.86	0.10471

Looking at the combined point estimates, we can see that for both the 14‐day and 28‐day regimens, the estimate based on Tippett's method is the smallest (i.e., most anti‐conservative for alternative = “less”), while the estimate based on the two‐trials rule is the largest (i.e., the most conservative). A similar but slightly attenuated pattern is seen for Fisher's (anti‐conservative) and Pearson's (conservative) methods, whereas the estimates from meta‐analysis and Edgington's method are almost identical and fall somewhere between the individual trials' effect estimates. All point estimates are thus consistent with the theoretically expected behavior of the methods.

It is interesting to consider the median estimate as a weighted average of the trial‐specific point estimates, and to determine the corresponding (implicit) weights. Table 3 reports the weight of the point estimate from RESPIRE 1 toward the median estimate (the weight from RESPIRE 2 is one minus the weight from RESPIRE 1). The more extreme estimate (RESPIRE 1 in the 14‐day group, and RESPIRE 2 in the 28‐day group) contributes more to Tippett's and Fisher's estimates and less to Pearson's and the two‐trials rule estimates, which align with the expected behavior. Similarly, the weight of RESPIRE 1 is slightly larger for Edgington's method than meta‐analysis because Edgington's estimate gives more weight to trials with larger standard errors due to its inverse standard error weighting.

Looking at the CIs, we can see that meta‐analysis produces narrower CIs than the other methods for both treatment regimens. The widest CIs are produced by Edgington's method. For the 28‐day regimen, Edgington's 95% CI is the only method that includes both trial effect estimates, and as a result is even wider than the CIs from the individual trials, reflecting the apparent heterogeneity. Looking at the decision based on the CIs, we can see that for the 14‐day regimens, all 95% CIs exclude a log rate ratio of zero, the value of no effect, while all 99.875% CIs include it. However, for the 28‐day regimens, the 95% CIs from meta‐analysis, Fisher's, and Tippett's methods exclude zero. The other method's 95% CIs include zero, but only Edgington's method also includes the point estimate from RESPIRE 2. Finally, the 99.875% CIs of all methods include zero, thus leading to identical decisions at the one‐sided $0.025^{2}=0.000625$ level. Note that for each method, the decision based on the CI is compatible with the combined *p*‐values in Table 3, for example, a 99.875% CI excludes a log rate ratio of zero only if also the combined one‐sided *p*‐value is less than 0.000625.

### 4.2 The ORBIT Trials

**FIGURE 4:**
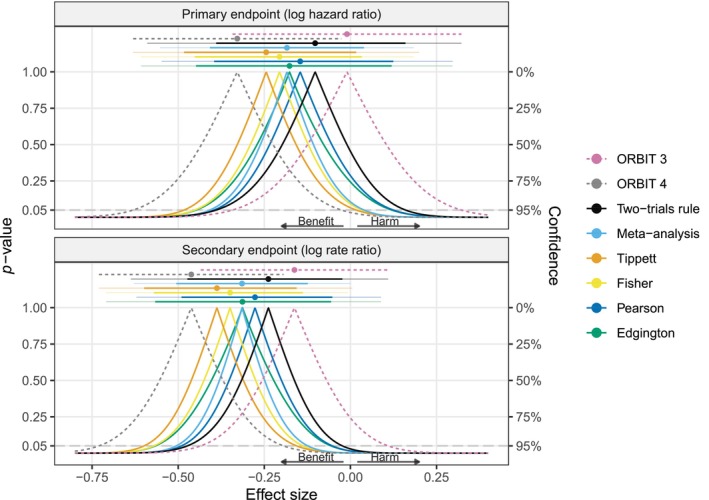
Results of the ORBIT trials [41] for the effect of ciprofloxacin in patients with non‐cystic fibrosis bronchiectasis and chronic lung infection with *Pseudomonas aeruginosa*. The two‐sided *p*‐value functions of the individual trials (dashed lines), and the combined *p*‐value functions (solid lines) based on the two‐trials rule, fixed‐effect meta‐analysis, Tippett's, Fisher's, Pearson's, and Edgington's methods are shown along with corresponding median estimates and CIs (95% and 99.875% via telescope lines). All *p*‐values are based on the alternative “less” and then converted to two‐sided *p*‐values via the centrality function 2min{p,1−p}.

Another pair of clinical trials that investigated the effect of ciprofloxacin are the ORBIT 3 and ORBIT 4 trials [41]. The trials assessed the effect of inhaled liposomal ciprofloxacin compared to placebo in patients with non‐cystic fibrosis bronchiectasis and chronic lung infection with *Pseudomonas aeruginosa*. Like the RESPIRE trials, the ORBIT trials also showed considerable heterogeneity. Figure 4 shows *p*‐values, point estimates, and CIs for the primary endpoint (time to the first exacerbation; effect quantified with a log hazard ratio) and a secondary endpoint (frequency of exacerbations; effect quantified with a log rate ratio). Table 4 gives numerical summaries.

**TABLE 4:** Point estimates (with implicit weights), 95% CIs (with widths), and *p*‐values for the ORBIT trials [41]. Note that weights and CI widths are computed from unrounded numbers and may not always exactly correspond to the rounded point estimates and CIs.

	Log rate ratio	Weight ORBIT 3	95% CI	CI width	*P*‐value (one‐sided)
Primary endpoint (log hazard ratio)					
ORBIT 3	−0.01		−0.34 to 0.32	0.66	0.47636
ORBIT 4	−0.33		−0.63 to −0.03	0.60	0.01657
Two‐trials rule	−0.10	0.71	−0.39 to 0.16	0.55	0.22692
Meta‐analysis	−0.18	0.45	−0.41 to 0.04	0.45	0.05305
Tippett	−0.24	0.26	−0.48 to 0.02	0.50	0.03286
Fisher	−0.21	0.39	−0.45 to 0.03	0.49	0.04610
Pearson	−0.15	0.57	−0.40 to 0.12	0.52	0.14328
Edgington	−0.18	0.48	−0.45 to 0.12	0.57	0.12149
Secondary endpoint (log rate ratio)					
ORBIT 3	−0.16		−0.43 to 0.11	0.54	0.12083
ORBIT 4	−0.46		−0.73 to −0.19	0.54	0.00036
Two‐trials rule	−0.24	0.75	−0.47 to −0.02	0.45	0.01460
Meta‐analysis	−0.31	0.49	−0.51 to −0.12	0.38	0.00062
Tippett	−0.39	0.25	−0.60 to −0.16	0.44	0.00072
Fisher	−0.35	0.38	−0.57 to −0.14	0.43	0.00048
Pearson	−0.28	0.62	−0.49 to −0.05	0.44	0.00765
Edgington	−0.31	0.50	−0.57 to −0.06	0.51	0.00734

We see that there is substantial heterogeneity for the primary endpoint, with the point estimate from ORBIT 3 close to zero ($\log\hat{\text{HR}}=-0.01$ with 95% CI from −0.34 to 0.32), whereas the estimate from ORBIT 4 indicates a more beneficial treatment effect ($\log\hat{\text{HR}}=-0.33$ with 95% CI from −0.63 to −0.03). While the theoretically expected patterns of the different median estimates and CIs are visible, the qualitative decisions based on all the different combination methods are the same at both the 0.025 and $0.025^{2}$ levels.

Looking at the secondary endpoint, there is also considerable heterogeneity between the results from ORBIT 3 ($\log\hat{\text{RR}}=-0.16$ with 95% CI from −0.43 to 0.11) and ORBIT 4 ($\log\hat{\text{RR}}=-0.46$ with 95% CI from −0.73 to −0.19) leading to some more noticeable qualitative differences between the methods. That is, the 99.875% CIs from meta‐analysis and Fisher's method excludes a log rate ratio of zero while the remaining methods include it, leading to different decisions at the $0.025^{2}$ level. Again, Edgington's CI is much wider than the others due to the substantial heterogeneity.

In summary, the analyses of the RESPIRE and ORBIT trials showed how combined *p*‐value functions allow us to obtain point estimates, CIs, and *p*‐values that are inherently compatible. They also showed that different combination methods can lead to different inferences and decisions, especially in the presence of between‐trial heterogeneity, highlighting the need to think about the estimand of interest.

## 5 Extension to More Than Two Trials

The methods discussed so far have focused on the setting where only two trials are available, but in practice, it may happen that investigators want to assess the combined evidence from more than two trials. In this context, Rosenkranz [32] suggested that decision rules should maintain the type I error rate of the two‐trials rule for two studies $\alpha^{2}$, even if there are more than two studies. This can be implemented using combined *p*‐value functions, as all methods considered before can be generalized to more than two trials [13, 28]. A decision rule can then be based on the combined one‐sided *p*‐value for the null hypothesis of no effect or a $(1-2\alpha^{2})\times 100\%$ CI obtained from a combined *p*‐value function. In addition, a point estimate and 95% CI can be used to summarize the combined evidence.

While all point estimates and CIs in this setting can be computed numerically, some of the analytical results derived earlier generalize to more than two studies. Specifically, closed‐form median estimates and CIs remain available for the two‐trials rule, Tippett's method, and meta‐analysis, whereas such closed‐form solutions are not available for Fisher's, Pearson's, and Edgington's methods [28]. In particular, for Edgington's method, one might expect the median estimate (18) to generalize by incorporating additional effect estimates with inverse standard error weights. However, a comparison with numerically computed median estimates showed that this is not the case. Thus, the inverse standard error weighted average in Equation (18) corresponds to Edgington's median estimate only in the setting of two trials.

**FIGURE 5:**
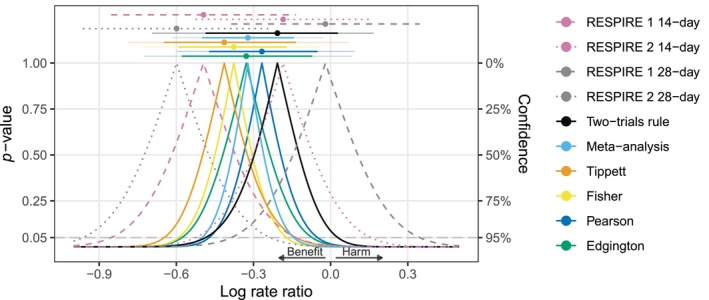
Combined *p*‐value function based on all four estimates from the RESPIRE trials [6, 7, 8] along with corresponding median estimates and CIs (95% and 99.875% via telescope lines).

Figure 5 shows *p*‐value functions that combine all four results from the two RESPIRE trials, as also done by Chotirmall and Chalmers [8] with fixed‐effect meta‐analysis. Looking at the median estimates, we see the same patterns as when the methods are applied to only two trials; The median estimates from meta‐analysis and Edgington's method are somewhere in between the trials' individual estimates. The median estimates based on Fisher's and Tippett's method are the most anti‐conservative, and the median estimates based on Pearson's method and the two‐trials rule are the most conservative. A decision rule that maintains the $\alpha^{2}=0.025^{2}$ type I error rate of the two trials rule could now be defined by flagging drug efficacy when the $(1-2\alpha^{2})\times 100\%=99.875\%$ excludes the null value. Following this rule, we can see that Fisher's method and meta‐analysis flag efficacy while the remaining methods do not. In sum, this example illustrates how combined *p*‐value functions can be applied to more than two trials, while allowing to maintain the same type I error rate as for two trials.

## 6 Discussion

The two‐trials rule has been widely discussed in the literature, but discussions have mostly focused on hypothesis testing characteristics, such as power or type I error rate [3, 4, 5, 9, 10, 22, 30, 31, 32, 36, 42, 43, 44]. In this paper, we took a different perspective, systematically examining the two‐trials rule and alternative methods in terms of effect estimation. By casting them in a combined *p*‐value function framework, we derived compatible *p*‐values, confidence intervals, and point estimates. These quantities are compatible in the sense that the two‐sided *p*‐value for a null value is less than α if and only if the null value is excluded by the (1−α)×100% CI, and the point estimate is contained in the CI at any confidence level. While meta‐analytic effect estimates, CIs, and *p*‐values have been well studied, our novel results enable computation of CIs and effect estimates based on the two‐trials rule. Investigators could therefore report not only individual trial *p*‐values (essentially the two‐trials rule) and meta‐analytic estimates, but also point estimates and CIs based on the two‐trials rule.

Our findings also clarify how different *p*‐value combination methods implicitly target different estimands. Reassuringly, under effect homogeneity (i.e., the same true effect in both trials), all methods yield consistent point estimates and CIs that shrink toward the true effect as standard errors decrease, although some show bias. Theoretically, meta‐analysis has the smallest variance among all unbiased estimators (attaining the Cramér‐Rao lower bound) and may be preferred. However, under effect heterogeneity—arguably the more realistic scenario—it is less clear which method should be recommended. The two‐trials rule and Pearson's method are conservative (targeting the less extreme effect), Fisher's and Tippett's methods are anti‐conservative (targeting the more extreme effect), while Edgington's method and meta‐analysis are balanced (targeting a weighted average). This raises an important question: What kind of effect is of scientific interest when the true trial effects differ? If the investigators are interested in the less extreme effect—arguably a sensible choice when the effects relate to a medical treatment with potential side effects—then the two‐trials rule and Pearson's method seem reasonable. On the other hand, a weighted average effect, as targeted by meta‐analysis and Edgington's method, might be a relevant estimand if it is representative for a larger population [34]. Finally, the more extreme effect might be the relevant estimand if the maximum achievable benefit of a treatment is of scientific interest, in which case Tippett's and Fisher's methods might be reasonable choices. This parallels the findings of Heard and Rubin‐Delanchy [21], who showed that many *p*‐value combination methods are equivalent to a likelihood ratio test for specific alternative hypotheses. This means that each such method can be most powerful under certain conditions. Therefore, researchers must carefully reflect which alternative hypothesis is most relevant to their application—just as they need to reflect on choosing an appropriate estimand—to select a suitable combination method.

Beyond theoretical considerations, practical issues must be addressed. A major concern is that if the effect estimates from both trials are the same, the two‐trials rule, Tippett's, Fisher's, and Pearson's methods all produce counterintuitive effect estimates that differ from the one observed in both trials. Such point estimates are unintuitive and difficult to communicate to non‐statisticians. Moreover, only Edgington's method and meta‐analysis produce the same combined estimate and two‐sided confidence interval in case the alternative of the combined p‐values is changed [28], which seems another practically desired property. From this perspective, Edgington's method and meta‐analysis may be preferable. In particular, Edgington's method can also account for effect heterogeneity by widening its CI when there is heterogeneity and asymptotically always includes both effects. However, this is traded off with a less efficient median estimate under effect homogeneity, whose standard error can even be larger than those from both trials if they greatly differ. Finally, another practical challenge is aligning decisions based on a one‐sided combined *p*‐value thresholded at $\alpha^{2}$ with two‐sided CIs. This requires using a $(1-2\alpha^{2})\times 100\%$ confidence level. However, in many fields, researchers are not used to such confidence levels, so we suggest reporting both a more conventional level (e.g., 95%) along with $(1-2\alpha^{2})\times 100\%$ via telescope‐style CIs, as well as the underlying *p*‐value function, as in Figures 3, 4, 5. The idea of a telescope‐style CIs is not new but has been suggested before in different contexts [45].

A broader issue is the question of whether two trials are actually necessary. If the designs of the two trials are so similar that they can be considered exchangeable (“direct replications” [46]), there are various arguments in favor of conducting one large trial instead of two smaller ones [9, 10]. Also, our study demonstrates that having two trials instead of one makes estimation more complicated. Conversly, if the trial designs differ significantly (“conceptual replications” [46], for example, if they use different endpoints or populations), achieving success in both trials may provide more robust evidence of treatment efficacy. From this perspective, it is sensible to design the trials differently to some extent [10]. However, there is perhaps a limit to how different the trials can be, as when there is too much heterogeneity, combining the effect estimates into a single number would no longer be meaningful.

Our results have broader implications beyond the two‐trial setting. Methods for combining *p*‐values are also used in adaptive trials, where they enable combination of stage‐wise *p*‐values [47, 48, 49]. Combined *p*‐value functions can be generalized to more than two studies, making them applicable to meta‐analysis [13, 28]. They can also be applied to replication and real‐world evidence studies, where the two‐trials rule (under different names such as significance criterion or vote‐counting) is used to assess the replicability of original findings [40, 50, 51]. In all these scenarios, we may consider combined *p*‐value functions for parameter estimation, but in each application, researchers must also decide which combination method has the statistical properties to estimate the scientific effect of interest. Future research may also examine other combination methods beyond the ones considered here, such as the inverse chi‐square method [17, 52], the harmonic mean $\chi^{2}$ test [53], the Cauchy combination test [54], random‐effects meta‐analysis [33], and combining *p*‐value functions that are based on the exact distribution of the data rather than normality, for example, the *p*‐value function based on Fisher's exact test with mid‐*p* correction [28, 55]. Additionally, fixed‐effect meta‐analysis has a Bayesian interpretation, corresponding to posterior inferences assuming equal true study effects and a flat prior distribution. Investigating whether other *p*‐value combination methods have similar Bayesian justifications could be an interesting avenue for future work. To sum up, combined *p*‐value functions provide a unified approach for combining results from two trials that can be further developed theoretically. Moreover, our software implementation allows researchers to conveniently apply these methods in practice.

## Conflicts of Interest

The authors declare no conflicts of interest.

## Figures and Tables

**FIGURE B1:**
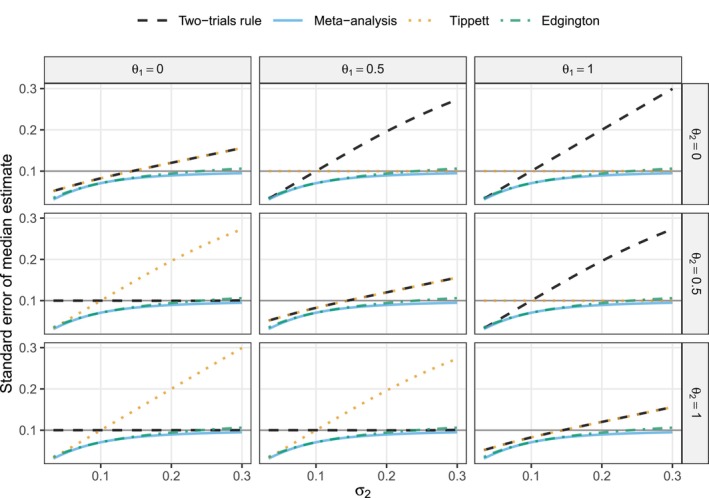
Comparison of median estimate standard errors across different scenarios of true trial effects $\theta_{1}$ and $\theta_{2}$ and trial standard error from the second trial $\sigma_{2}$. The standard error of the first trial is $\sigma_{1}=0.1$ across all scenarios. The standard errors from Fisher's and Pearson's methods are close to Tippett's method and the two‐trials rule, and are not shown to make the figure easier to read.

**FIGURE B2:**
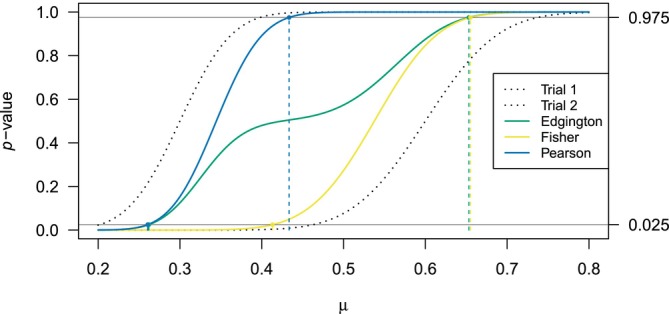
Two well‐separated *p*‐value functions (with alternative “greater”) and the associated combined *p*‐value functions based on Fisher's, Pearson's, and Edgington's methods. The dashed vertical lines and points denote the 95% CI limits computed with the approximate combined estimation functions (B5), (B6), and (B7). The effect estimates are ${\hat{\theta }}_{1}=0.3$ and ${\hat{\theta }}_{2}=0.6$ while the standard errors are $\sigma_{1}=0.05$ and $\sigma_{2}=0.07$.

## Data Availability

Data from the RESPIRE trials were extracted from Table 3 in De Soyza et al. [7] and Table 3 in Aksamit et al. [6]. Data from the ORBIT trials were extracted from page 219 in Haworth et al. [41]. Code and data to reproduce all numbers, tables, and figures are openly available at https://github.com/SamCH93/twotrials. Spreadsheets containing the numbers from Tables 3 and 4 with higher precision are also available at the repository. A snapshot of the repository at the time of writing is available at https://doi.org/10.5281/zenodo.15017483. The R package twotrials implementing combined *p*‐value function inference for two trials is available at https://doi.org/10.32614/CRAN.package.twotrials. We used the statistical programming language R version 4.4.1 (June 14, 2024) for analyses [56] along with the confMeta [57], ggplot2 [58], kableExtra [59], dplyr [60], and knitr [61] packages.

## Appendix A The R Package Twotrials

We have developed the twotrials R package for easily conducting combined *p*‐value function inference based on the parameter estimates (with standard errors) from two trials. The package can be installed from the Comprehensive R Archive Network (CRAN) via the R command install.packages(“twotrials”).

For every *p*‐value combination method discussed in this paper, the package provides a combined *p*‐value function (e.g., pEdgington) and a combined estimation function (e.g., muEdgington). While these can be used to manually compute *p*‐values and parameter estimates, the convenience function twotrials automatically computes estimates and *p*‐values based on all methods, and allows for easy printing and plotting of the results. The following code chunk illustrates its usage by reproducing the results for the 14‐day treatment group from Table 3.

Note that, for each combined estimate, the function also returns the weights $w_{1}$ (W1) and $w_{2}$ (W2). These represent the implicit linear weights of the point estimates from trials 1 and 2 towards the combined estimate, that is, $\hat{\mu }(1/2)=w_{1}{\hat{\theta }}_{1}+w_{2}{\hat{\theta }}_{2}$. Such weights aid interpretation by indicating how close each trial estimate is to the combined estimate. Finally, applying the plot function to the resulting object makes it easy to display the combined *p*‐value functions, as demonstrated below.

## Appendix B Technical Details

This appendix contains technical details on the derivation of results from the main paper.

### Expectation of the Combined Estimation Function

Consider the random variables

(B1)
 X=min{θ^1+σ1q,θ^2+σ2q}and Y=max{θ^1−σ1q,θ^2−σ2q}

Table B1 shows that for certain choices of the constant q, X and Y are equal to the combined estimation functions from the two‐trials rule (3) and Tippett's method (12) in the main paper, and the approximate combined estimation functions from Fisher's (B5), Pearson's (B6), and Edgington's methods (B7) and (B8) discussed below. Note that for Edgington's method (with a=1/2) and meta‐analysis, the distribution of the combined estimation function is normal and its expectation is therefore known and need not be derived here.

**TABLE B1:** Constants q for Which X or Y Are Equal to the Combined Estimation Function of a Specific Method.

Method	Alternative “greater”	Alternative “less”
Two‐trials rule	X with $q=z_{\sqrt{a}}$	Y with $q=z_{\sqrt{a}}$
Tippett	Y with $q=z_{\sqrt{1-a}}$	X with $q=z_{\sqrt{1-a}}$
Fisher (approximate)	Y with $q=-z_{\exp\{-\chi_{4}^{2}(1-a)/2\}}$	X with $q=-z_{\exp\{-\chi_{4}^{2}(1-a)/2\}}$
Pearson (approximate)	X with $q=-z_{\exp\{-\chi_{4}^{2}(a)/2\}}$	Y with $q=-z_{\exp\{-\chi_{4}^{2}(a)/2\}}$
Edgington (approximate, a<1/2)	X with $q=z_{\sqrt{2a}}$	Y with $q=z_{\sqrt{2a}}$
Edgington (approximate, a>1/2)	Y with $q=z_{\sqrt{2(1-a)}}$	X with $q=z_{\sqrt{2(1-a)}}$

According to the assumptions stated at the beginning of Section 2, ${\hat{\theta }}_{1}$ and ${\hat{\theta }}_{2}$ are independent normal random variables with means $\theta_{1},\theta_{2}$ and variances $\sigma_{1}^{2},\sigma_{2}^{2}$. We can therefore use the results from Nadarajah and Kotz [62] on closed‐form expressions for the moments of minima and maxima of bivariate Gaussian random vectors. That is, using their Equations (9) and (11), it can be shown that the expectations of X and Y are given by

$$\begin{array}{ll} E(X) & =(\theta_{1}+\sigma_{1}\;q)\times \Phi \left(\frac{\theta_{2}-\theta_{1}+q(\sigma_{2}-\sigma_{1})}{\sqrt{\sigma_{1}^{2}+\sigma_{2}^{2}}}\right)\\ & \;+(\theta_{2}+\sigma_{2}\;q)\times \Phi \left(\frac{\theta_{1}-\theta_{2}+q(\sigma_{1}-\sigma_{2})}{\sqrt{\sigma_{1}^{2}+\sigma_{2}^{2}}}\right)\\ & \;-\sqrt{\sigma_{1}^{2}+\sigma_{2}^{2}}\times \phi \left(\frac{\theta_{2}-\theta_{1}+q(\sigma_{2}-\sigma_{1})}{\sqrt{\sigma_{1}^{2}+\sigma_{2}^{2}}}\right) \end{array}$$

and

$$\begin{array}{ll} E(Y) & =(\theta_{1}-\sigma_{1}\;q)\times \Phi \left(\frac{\theta_{1}-\theta_{2}+q(\sigma_{2}-\sigma_{1})}{\sqrt{\sigma_{1}^{2}+\sigma_{2}^{2}}}\right)\\ & \;+(\theta_{2}-\sigma_{2}\;q)\times \Phi \left(\frac{\theta_{2}-\theta_{1}+q(\sigma_{1}-\sigma_{2})}{\sqrt{\sigma_{1}^{2}+\sigma_{2}^{2}}}\right)\\ & \;+\sqrt{\sigma_{1}^{2}+\sigma_{2}^{2}}\times \phi \left(\frac{\theta_{1}-\theta_{2}+q(\sigma_{2}-\sigma_{1})}{\sqrt{\sigma_{1}^{2}+\sigma_{2}^{2}}}\right) \end{array}$$

respectively. The expectation of the combined estimation function from a specific method is thus obtained by setting the constant q to the corresponding value. For example, the expectation of the median estimate (a=1/2) from the two‐trials rule with alternative “greater” and $\sigma_{1}=\sigma_{2}=\sigma$ in Equation (6) is obtained from E(X) with $q=z_{\sqrt{1/2}}$.

### Median Estimate Standard Errors

Since the median estimates from the meta‐analysis and Edgington's method are simple linear combinations of the trial effect estimates, their standard errors can be straightforwardly derived to be

$$\begin{array}{lllllll} & \sigma_{\text{MA}}=\frac{1}{\sqrt{1/\sigma_{1}^{2}+1/\sigma_{2}^{2}}}\; & & \text{and}\; & & \sigma_{E}=\frac{\sqrt{2}}{1/\sigma_{1}+1/\sigma_{2}}\; & \end{array}$$

By applying algebraic manipulations to $\sigma_{\text{MA}}\leq \sigma_{E}$, one can see that the meta‐analytic standard error is never larger than Edgington's standard error, with equality if and only if the trial standard error coincide ($\sigma_{1}=\sigma_{2}$). Similarly, by applying algebraic manipulations to $\sigma_{E}\leq \sigma_{1}$ and $\sigma_{E}\leq \sigma_{2}$, one can see that Edgington's standard error is only equal to or smaller than either trial standard error if $\sqrt{2}-1\leq \sigma_{2}/\sigma_{1}\leq 1/(\sqrt{2}-1)=\sqrt{2}+1$.

For the remaining methods, the (approximate) median estimates are given by X and Y in Equation (B1) and Table B1 with a=1/2. We can therefore use the results from Nadarajah and Kotz [62] to obtain their second moments

$$\begin{array}{ll} E(X^{2}) & =\{\sigma_{1}^{2}+{\left(\theta_{1}+\sigma_{1}\;q\right)}^{2}\}\;\Phi \left(\frac{\theta_{2}-\theta_{1}+q(\sigma_{2}-\sigma_{1})}{\sqrt{\sigma_{1}^{2}+\sigma_{2}^{2}}}\right)\\ & \;+\{\sigma_{2}^{2}+{\left(\theta_{2}+\sigma_{2}\;q\right)}^{2}\}\;\Phi \left(\frac{\theta_{1}-\theta_{2}+q(\sigma_{1}-\sigma_{2})}{\sqrt{\sigma_{1}^{2}+\sigma_{2}^{2}}}\right)\\ & \;-\{\theta_{1}+\theta_{2}+q(\sigma_{1}+\sigma_{2})\}\sqrt{\sigma_{1}^{2}+\sigma_{2}^{2}}\\ & \;\times \phi \left(\frac{\theta_{2}-\theta_{1}+q(\sigma_{2}^{2}-\sigma_{1}^{2})}{\sqrt{\sigma_{1}+\sigma_{2}}}\right) \end{array}$$

and

$$\begin{array}{ll} E(Y^{2}) & =\{\sigma_{1}^{2}+{\left(\theta_{1}-\sigma_{1}\;q\right)}^{2}\}\;\Phi \left(\frac{\theta_{1}-\theta_{2}+q(\sigma_{2}-\sigma_{1})}{\sqrt{\sigma_{1}^{2}+\sigma_{2}^{2}}}\right)\\ & \;+\{\sigma_{2}^{2}+{\left(\theta_{2}-\sigma_{2}\;q\right)}^{2}\}\;\Phi \left(\frac{\theta_{2}-\theta_{1}+q(\sigma_{1}-\sigma_{2})}{\sqrt{\sigma_{1}^{2}+\sigma_{2}^{2}}}\right)\\ & \;+\{\theta_{1}+\theta_{2}-q(\sigma_{1}+\sigma_{2})\}\sqrt{\sigma_{1}^{2}+\sigma_{2}^{2}}\\ & \;\times \phi \left(\frac{\theta_{1}-\theta_{2}+q(\sigma_{2}-\sigma_{1})}{\sqrt{\sigma_{1}^{2}+\sigma_{2}^{2}}}\right) \end{array}$$

and corresponding standard errors. For example, assuming equal standard errors ($\sigma_{1}=\sigma_{2}=\sigma$), equal true effects ($\theta_{1}=\theta_{2}=\theta$), and the alternative “greater”, the standard error of the two‐trials rule median estimate (X with $q=z_{\sqrt{1/2}}$) simplifies to (7).

Figure B1 shows the standard errors from the median estimates of the two‐trials rule, meta‐analysis, Tippett's, and Edgington's methods for various scenarios of true trial effects and trial standard errors that should resemble typical ranges for standardized mean difference parameters. The approximate standard errors from Fisher's and Pearson's methods are close to the ones from Tippett's method and the two‐trials rule, and therefore not shown to make the plot easier to read.

We can see that the standard error from meta‐analysis is always the lowest and is always smaller than the minimum of the two standard errors ($\sigma_{\text{MA}}\leq \min\{\sigma_{1},\sigma_{2}\}$). The standard error from Edgington's method is equal (when $\sigma_{1}=\sigma_{2}$) to or larger than the meta‐analytic one, and can exceed the minimum standard error from the two trials (e.g., for $\sigma_{1}=0.1$ and $\sigma_{2}=0.3$, it is $\sigma_{E}=0.106$). The standard errors for both Edgington's method and meta‐analysis only depend on the trials' standard errors, but not on the true effects, so their standard errors are the same across all panels in Figure B1. This is not the case for the two‐trials rule and Tippett's method, which show a more irregular behavior. When the true trial effects coincide ($\theta_{1}=\theta_{2}$; panels on the diagonal), the combined standard error decreases with decreasing standard error from the second trial $\sigma_{2}$. However, when the true effect from the first trial is smaller than that one from the second trial ($\theta_{1}<\theta_{2}$; upper off‐diagonal panels), the combined standard error from Tippett's method remains constant, whereas the standard error from the two‐trials rule changes drastically with changing $\sigma_{2}$. The opposite occurs when the effect from the first trial is larger ($\theta_{1}>\theta_{2}$; lower off‐diagonal panels). This is plausible, as these methods target the minimum (two‐trials rule) or maximum (Tippett) effect, meaning that the standard error of the trial with the minimum or maximum effect mainly affects the standard error of the combined estimate.

### Limiting Combined Estimation Functions

Consider again the random variables X and Y as defined in Equation (B1). Their cumulative distribution functions can be derived to be

$$\begin{array}{ll} \mathrm{Pr}(X\leq x) & =\mathrm{Pr}(\min\{{\hat{\theta }}_{1}+\sigma_{1}\;q,{\hat{\theta }}_{2}+\sigma_{2}\;q\}\leq x)\\ & =1-\mathrm{Pr}({\hat{\theta }}_{1}+\sigma_{1}\;q>x,{\hat{\theta }}_{2}+\sigma_{2}\;q>x)\\ & =1-\{\mathrm{Pr}({\hat{\theta }}_{1}+\sigma_{1}\;q>x)\times \mathrm{Pr}({\hat{\theta }}_{2}+\sigma_{2}\;q>x)\}\\ & =1-\left\{\Phi \left(\frac{\theta_{1}-x}{\sigma_{1}}+q\right)\times \Phi \left(\frac{\theta_{2}-x}{\sigma_{2}}+q\right)\right\} \end{array}$$

and

$$\begin{array}{ll} \mathrm{Pr}(Y\leq y) & =\mathrm{Pr}(\max\{{\hat{\theta }}_{1}-\sigma_{1}\;q,{\hat{\theta }}_{2}-\sigma_{2}\;q\}\leq y)\\ & =\mathrm{Pr}({\hat{\theta }}_{1}-\sigma_{1}\;q\leq y,{\hat{\theta }}_{2}-\sigma_{2}\;q\leq y)\\ & =\mathrm{Pr}({\hat{\theta }}_{1}-\sigma_{1}\;q\leq y)\times \mathrm{Pr}({\hat{\theta }}_{2}-\sigma_{2}\;q\leq y)\\ & =\Phi \left(\frac{y-\theta_{1}}{\sigma_{1}}+q\right)\times \Phi \left(\frac{y-\theta_{2}}{\sigma_{2}}+q\right) \end{array}$$

Letting the standard errors $\sigma_{1}$ and $\sigma_{2}$ go to zero, this leads to

(B2)
$$\begin{array}{ll} \underset{\sigma_{1},\sigma_{2}↓0}{\lim}\mathrm{Pr}(X\leq x) & =1-\{1_{\left(-\infty ,\theta_{1}\right)}(x)\times 1_{\left(-\infty ,\theta_{2}\right)}(x)\}\\ & =1_{\left[\min\{\theta_{1},\theta_{2}\},+\infty \right)}(x) \end{array}$$

and

(B3)
$$\begin{array}{ll} \underset{\sigma_{1},\sigma_{2}↓0}{\lim}\mathrm{Pr}(Y\leq y) & =1_{\left[\theta_{1},+\infty \right)}(y)\times 1_{\left[\theta_{2},+\infty \right)}(y)\\ & =1_{\left[\max\{\theta_{1},\theta_{2}\},+\infty \right)}(y) \end{array}$$

where $1_{A}(x)=1$ if x∈A and 0 otherwise, and the constant q vanishes. Since (B2) and (B3) is the cumulative distribution function of a degenerate random variable at $\min\{\theta_{1},\theta_{2}\}$ and $\max\{\theta_{1},\theta_{2}\}$, respectively, this implies that the combined estimation functions given by X and Y converge in probability to $\min\{\theta_{1},\theta_{2}\}$ and $\max\{\theta_{1},\theta_{2}\}$, respectively, for any constant q. Thus, all combined estimation functions from Table B1 converge in probability to $\min\{\theta_{1},\theta_{2}\}$ or $\max\{\theta_{1},\theta_{2}\}$ as $\sigma_{1}$ and $\sigma_{2}$ decrease.

### Approximate Combined Estimation Functions

Suppose that the trials' individual *p*‐value functions

$$\begin{array}{lll} & p_{1}(\mu )=\left\{\begin{array}{ll} 1-\Phi \left(\frac{{\hat{\theta }}_{1}-\mu }{\sigma_{1}}\right)\; & \text{for alternative = “greater”}\\ \Phi \left(\frac{{\hat{\theta }}_{1}-\mu }{\sigma_{1}}\right)\; & \text{for alternative “less”} \end{array}\right.\; & \\ & p_{2}(\mu )=\left\{\begin{array}{ll} 1-\Phi \left(\frac{{\hat{\theta }}_{2}-\mu }{\sigma_{2}}\right)\; & \text{for alternative = “greater”}\\ \Phi \left(\frac{{\hat{\theta }}_{2}-\mu }{\sigma_{2}}\right)\; & \text{for alternative = “less”} \end{array}\right.\; & \end{array}$$

are “well‐separated” in the sense that in the region where $p_{1}(\mu )$ changes from 0 to 1, $p_{2}(\mu )$ stays almost constant at 0 or 1, see the dotted lines in Figure B2 for an example. This happens when either the estimates ${\hat{\theta }}_{1}$ and ${\hat{\theta }}_{2}$ are far apart and/or the standard errors $\sigma_{1}$ and $\sigma_{2}$ are small relative to the estimates (provided the estimates are not equal). Note that asymptotically, the individual *p*‐value functions approach the step functions

$$\begin{array}{lll} & \lim_{\sigma_{1}↓0}p_{1}(\mu )=\left\{\begin{array}{ll} 1_{\left[\theta_{1},+\infty \right)}(\mu )\; & \text{for alternative = “greater”}\\ 1_{\left(-\infty ,\theta_{1}\right]}(\mu )\; & \text{for alternative = “less”} \end{array}\right. & \\ & \lim_{\sigma_{2}↓0}p_{2}(\mu )=\left\{\begin{array}{ll} 1_{\left[\theta_{2},+\infty \right)}(\mu )\; & \text{for alternative = “greater”}\\ 1_{\left(-\infty ,\theta_{2}\right]}(\mu )\; & \text{for}\text{alternative = “less”} \end{array}\right. & \end{array}$$

Hence, with decreasing standard errors, the trials' *p*‐value functions eventually become well‐separated whenever the true effects $\theta_{1}$ and $\theta_{2}$ are unequal.

In case of well‐separated *p*‐value functions, we can approximate the combined *p*‐value function from Fisher's, Pearson's, and Edgington's method by setting one of the *p*‐values to 0 or 1, depending on the alternative and combination method, and derive an approximate but closed‐form combined estimation function. For example, in Figure B2 the combined *p*‐value from Fisher's method (13) remains virtually constant for increasing μ when the first individual *p*‐function increases and only starts to increase as the second *p*‐value function increases.

We may hence approximate Fisher's combined *p*‐value by

(B4)
$$\begin{array}{ll} p_{F}(\mu ) & =\left\{\begin{array}{ll} 1-\mathrm{Pr}\left[\chi_{4}^{2}\leq -2\log\left\{1-\Phi \left(\max\left\{\frac{{\hat{\theta }}_{1}-\mu }{\sigma_{1}},\frac{{\hat{\theta }}_{2}-\mu }{\sigma_{2}}\right\}\right)\right\}\right]\; & \text{for alternative=“greater”}\\ 1-\mathrm{Pr}\left[\chi_{4}^{2}\leq -2\log\left\{\Phi \left(\min\left\{\frac{{\hat{\theta }}_{1}-\mu }{\sigma_{1}},\frac{{\hat{\theta }}_{2}-\mu }{\sigma_{2}}\right\}\right)\right\}\right]\; & \text{for}\text{alternative=“less”} \end{array}\right. \end{array}$$

The corresponding combined estimation function can then be obtained by equating (B4) to a and solving for μ, which leads to

(B5)
$${\hat{\mu }}_{F}(a)=\left\{\begin{array}{ll} \max\{{\hat{\theta }}_{1}+\sigma_{1}\;z_{\exp\{-\chi_{4}^{2}(1-a)/2\}},\; & \\ \;{\hat{\theta }}_{2}+\sigma_{2}\;z_{\exp\{-\chi_{4}^{2}(1-a)/2\}}\}\; & \text{for alternative = “greater”}\\ \min\{{\hat{\theta }}_{1}-\sigma_{1}\;z_{\exp\{-\chi_{4}^{2}(1-a)/2\}},\; & \\ \;{\hat{\theta }}_{2}-\sigma_{2}\;z_{\exp\{-\chi_{4}^{2}(1-a)/2\}}\}\; & \text{for alternative = “less”} \end{array}\right.$$

The dashed yellow vertical lines in Figure B2 show the limits of a 95% CI computed via (B5), demonstrating that the approximation is accurate in this case, despite the finite standard errors.

In an analogous fashion, the combined *p*‐value function based on Pearson's method can be approximated by

$$\begin{array}{ll} p_{P}(\mu ) & =\left\{\begin{array}{ll} \mathrm{Pr}\left[\chi_{4}^{2}\leq -2\log\left\{\Phi \left(\min\left\{\frac{{\hat{\theta }}_{1}-\mu }{\sigma_{1}},\frac{{\hat{\theta }}_{2}-\mu }{\sigma_{2}}\right\}\right)\right\}\right]\; & \text{for alternative=“greater”}\\ \mathrm{Pr}\left[\chi_{4}^{2}\leq -2\log\left\{1-\Phi \left(\max\left\{\frac{{\hat{\theta }}_{1}-\mu }{\sigma_{1}},\frac{{\hat{\theta }}_{2}-\mu }{\sigma_{2}}\right\}\right)\right\}\right]\; & \text{for}\text{alternative=“less”} \end{array}\right. \end{array}$$

leading to the approximate combined estimation function

(B6)
$${\hat{\mu }}_{P}(a)=\left\{\begin{array}{ll} \min\{{\hat{\theta }}_{1}-\sigma_{1}\;z_{\exp\{-\chi_{4}^{2}(a)/2\}},\; & \\ \;{\hat{\theta }}_{2}-\sigma_{2}\;z_{\exp\{-\chi_{4}^{2}(a)/2\}}\}\; & \text{for alternative = “greater”}\\ \max\{{\hat{\theta }}_{1}+\sigma_{1}\;z_{\exp\{-\chi_{4}^{2}(a)/2\}},\; & \\ \;{\hat{\theta }}_{2}+\sigma_{2}\;z_{\exp\{-\chi_{4}^{2}(a)/2\}}\}\; & \text{for alternative = “less”} \end{array}\right.$$

The functions (B5) and (B6) based on Fisher's and Pearson's methods have a striking similarity to the combined estimation functions based on Tippett's method (12) and the two‐trials rule (3), respectively, as they again involve shifted maxima/minima of the trial estimates.

Similarly, Edgington's combined *p*‐value function can be approximated by

$$\begin{array}{l} p_{E}(\mu )=\left\{\begin{array}{ll} {\left\{1-\Phi \left(\min\left\{\frac{{\hat{\theta }}_{1}-\mu }{\sigma_{1}},\frac{{\hat{\theta }}_{2}-\mu }{\sigma_{2}}\right\}\right)\right\}}^{2}/\;2\; & \text{if}\;\mu <\frac{{\hat{\theta }}_{1}/\sigma_{1}+{\hat{\theta }}_{2}/\sigma_{2}}{1/\sigma_{1}+1/\sigma_{2}}\\ \frac{1}{2}\; & \text{if}\;\mu =\frac{{\hat{\theta }}_{1}/\sigma_{1}+{\hat{\theta }}_{2}/\sigma_{2}}{1/\sigma_{1}+1/\sigma_{2}}\\ 1-{\left\{\Phi \left(\max\left\{\frac{{\hat{\theta }}_{1}-\mu }{\sigma_{1}},\frac{{\hat{\theta }}_{2}-\mu }{\sigma_{2}}\right\}\right)\right\}}^{2}/\;2\; & \text{else} \end{array}\right. \end{array}$$

for the alternative “greater” and with

$$\begin{array}{l} p_{E}(\mu )=\left\{\begin{array}{ll} {\left\{\Phi \left(\max\left\{\frac{{\hat{\theta }}_{1}-\mu }{\sigma_{1}},\frac{{\hat{\theta }}_{2}-\mu }{\sigma_{2}}\right\}\right)\right\}}^{2}/\;2\; & \text{if}\;\mu >\frac{{\hat{\theta }}_{1}/\sigma_{1}+{\hat{\theta }}_{2}/\sigma_{2}}{1/\sigma_{1}+1/\sigma_{2}}\\ \frac{1}{2}\; & \text{if}\;\mu =\frac{{\hat{\theta }}_{1}/\sigma_{1}+{\hat{\theta }}_{2}/\sigma_{2}}{1/\sigma_{1}+1/\sigma_{2}}\\ 1-{\left\{1-\Phi \left(\min\left\{\frac{{\hat{\theta }}_{1}-\mu }{\sigma_{1}},\frac{{\hat{\theta }}_{2}-\mu }{\sigma_{2}}\right\}\right)\right\}}^{2}/\;2\; & \text{else} \end{array}\right. \end{array}$$

for the alternative “less”. Consequently, the approximate combined estimation function is

(B7)
$${\hat{\mu }}_{E}(a)=\left\{\begin{array}{ll} \min\{{\hat{\theta }}_{1}+\sigma_{1}\;z_{\sqrt{2a}},{\hat{\theta }}_{2}+\sigma_{2}\;z_{\sqrt{2a}}\}\; & \text{for}\;a<1/2\\ \frac{{\hat{\theta }}_{1}/\sigma_{1}+{\hat{\theta }}_{2}/\sigma_{2}}{1/\sigma_{1}+1/\sigma_{2}}\; & \text{for}\;a=1/2\\ \max\{{\hat{\theta }}_{1}-\sigma_{1}\;z_{\sqrt{2(1-a)}},{\hat{\theta }}_{2}-\sigma_{2}\;z_{\sqrt{2(1-a)}}\}\; & \text{for}\;a>1/2 \end{array}\right.$$

for the alternative “greater” and

(B8)
$${\hat{\mu }}_{E}(a)=\left\{\begin{array}{ll} \max\{{\hat{\theta }}_{1}-\sigma_{1}\;z_{\sqrt{2a}},{\hat{\theta }}_{2}-\sigma_{2}\;z_{\sqrt{2a}}\}\; & \text{for}\;a<1/2\\ \frac{{\hat{\theta }}_{1}/\sigma_{1}+{\hat{\theta }}_{2}/\sigma_{2}}{1/\sigma_{1}+1/\sigma_{2}}\; & \text{for}\;a=1/2\\ \min\{{\hat{\theta }}_{1}+\sigma_{1}\;z_{\sqrt{2(1-a)}},{\hat{\theta }}_{2}+\sigma_{2}\;z_{\sqrt{2(1-a)}}\}\; & \text{for}\;a>1/2 \end{array}\right.$$

for the alternative “less”. The combined estimation functions (B7) and (B8) also include the closed‐form solution for the median estimate (a=1/2) from Equation (18) as this value does not require any approximation. Surprisingly, a (1−α)×100% CI with α<1/4 constructed from Equation (B7) or (B8) always includes the individual estimates ${\hat{\theta }}_{1}$ and ${\hat{\theta }}_{2}$ since the lower limit (a=α/2) is always smaller than the minimum of the two effect estimates, and the upper limit (a=1−α/2) is always larger than the maximum of the two. This demonstrates that Edgington's method reacts to heterogeneity by widening its CI to include both trial effect estimates.

All of these approximations become more accurate with decreasing standard errors as the individual *p*‐value functions become more separated. Since all approximate combined estimation functions ((B5), [Link], (B6), [Link], [Link], (B7), (B8)) are essentially shifted minima and maxima (apart from the median estimate of Edgington's method), the results from Appendix B.0.3 apply. That is, as the standard errors $\sigma_{1}$ and $\sigma_{2}$ decrease toward zero, all minima converge in probability to $\min\{\theta_{1},\theta_{2}\}$ while all maxima converge to $\max\{\theta_{1},\theta_{2}\}$.
